# Bottlenecks drive temporal and spatial genetic changes in alpine caddisfly metapopulations

**DOI:** 10.1186/1471-2148-11-278

**Published:** 2011-09-27

**Authors:** Lisa NS Shama, Karen B Kubow, Jukka Jokela, Christopher T Robinson

**Affiliations:** 1 Eawag, Swiss Federal Institute of Aquatic Science and Technology, 8600 Dübendorf, Switzerland, and Institute of Integrative Biology, ETH Zürich, Switzerland; 2 Leibniz-Institut for Marine Sciences IFM-GEOMAR, Düsternbrooker Weg 20, 24105 Kiel, Germany; 3 Alfred Wegener Institute for Polar and Marine Research, Wadden Sea Station Sylt, 25992 List, Germany

## Abstract

**Background:**

Extinction and re-colonisation of local populations is common in ephemeral habitats such as temporary streams. In most cases, such population turnover leads to reduced genetic diversity within populations and increased genetic differentiation among populations due to stochastic founder events, genetic drift, and bottlenecks associated with re-colonisation. Here, we examined the spatio-temporal genetic structure of 8 alpine caddisfly populations inhabiting permanent and temporary streams from four valleys in two regions of the Swiss Alps in years before and after a major stream drying event, the European heat wave in summer 2003.

**Results:**

We found that population turnover after 2003 led to a loss of allelic richness and gene diversity but not to significant changes in observed heterozygosity. Within all valleys, permanent and temporary streams in any given year were not differentiated, suggesting considerable gene flow and admixture between streams with differing hydroperiods. Large changes in allele frequencies after 2003 resulted in a substantial increase in genetic differentiation among valleys within one to two years (1-2 generations) driven primarily by drift and immigration. Signatures of genetic bottlenecks were detected in all 8 populations after 2003 using the *M*-ratio method, but in no populations when using a heterozygosity excess method, indicating differential sensitivity of bottleneck detection methods.

**Conclusions:**

We conclude that genetic differentiation among *A. uncatus *populations changed markedly both temporally and spatially in response to the extreme climate event in 2003. Our results highlight the magnitude of temporal population genetic changes in response to extreme events. More specifically, our results show that extreme events can cause rapid genetic divergence in metapopulations. Further studies are needed to determine if recovery from this perturbation through gradual mixing of diverged populations by migration and gene flow leads to the pre-climate event state, or whether the observed changes represent a new genetic equilibrium.

## Background

Fluctuations in population size are common ecological phenomena in all species that use patchy habitats. In metapopulation theory, subdivided groups of local populations are thought to persist in a balance between migration, extinction and re-colonisation - that is, local populations turnover at a specific rate that is determined by patch structure, metapopulation size and density of patches [1,2]. The frequency of population turnover can largely determine the degree and distribution of genetic variation in the metapopulation, and therefore have direct impacts on the evolutionary potential of local populations [3-5].

Population turnover, here defined as re-colonisation after catastrophic local extinction, often leads to genetic bottlenecks during colonisation (founder effects). Bottlenecks can cause a rapid loss of genetic variation, fixing mildly deleterious alleles and increasing the degree of inbreeding, thereby reducing the adaptive potential of the population [6-9]. More specifically, bottlenecks reduce genetic diversity through the loss of rare alleles and reduced heterozygosity, and also can change the distribution and temporal variance of allele frequencies [9,10]. In recently bottlenecked populations not yet in mutation-drift equilibrium, a transient (lasting only a few generations) excess of heterozygosity can occur because allelic diversity is reduced faster than heterozygosity [11-13]. In the long term, a reduction in heterozygosity is predicted if population size remains small [11]. However, immigration can increase the rate of genetic recovery after bottleneck events and is the most likely process maintaining high genetic diversity in populations that fluctuate in size [14].

In a metapopulation, local turnover can either increase or decrease genetic differentiation among local populations. Slatkin [4] argued that local extinction and re-colonisation dynamics imply ongoing gene flow, which will prevent local populations from becoming differentiated. However, whether or not populations become differentiated depends on the mode in which new populations are founded [5]. Population turnover will have the homogenizing effect predicted by Slatkin [4] when the number of colonisers is large and individuals originate from many source populations (migrant-pool model). In contrast, if the number of colonisers is small and individuals originate from only one or a few source populations (propagule-pool model), turnover can increase population differentiation [10,15]. The latter is more likely when turnover leads to a bottleneck, which reduces effective population size (N_e_) and accelerates genetic drift with different alleles being lost from each population. Recurring local extinctions will then limit the time available for subsequent gene flow to equalize allele frequencies [16,17]. Consequently, turnover can cause the spatial distribution of alleles (and population differentiation) to vary temporally [18-21].

Extinction and re-colonisation dynamics are common in species inhabiting ephemeral habitats. For example, populations in temporary streams and ponds are confronted with the selection pressure imposed by habitat drying and are prone to population turnover [22]. Populations can persist either by adapting to local conditions [1,23] or by recurring re-colonisation after local extinction [2,24]. Here, we examined the spatio-temporal genetic structure of alpine caddisfly (*Allogamus uncatus *Brauer) populations in the Swiss Alps. This species occurs in small, permanent and temporary streams above 600 m a.s.l. in the Alps, Carpathians and Balkan Peninsula. Populations are univoltine, surviving late summer dry stream periods as terrestrial adults [25]. Population extinctions regularly occur when temporary stream larvae do not complete development to emergence prior to stream drying (LNSS & CTR, unpublished observations). Previous life history studies of *A. uncatus *populations using common garden experiments showed no evidence for local (genetic) adaptation to hydroperiod regimes, and plastic responses to drying habitat cues were weak [26] or inconsistent among populations [27]. Consequently, these caddisflies likely maintain a population structure that conforms to the genetic metapopulation concept.

We investigated the genetic structure of populations in years before and after a major climate event, the European heat wave in summer 2003 [28]. Models of future climate predict not only a general warming, but also an increase in extreme weather events such as droughts [29]. Since alpine aquatic insects are already living in extreme environments, they may respond quickly to environmental change. Using permanent and temporary stream population pairs from several valleys in the Swiss Alps, we tested for evidence of population turnover and signatures of genetic bottlenecks in response to the extreme stream drying event of 2003, and then assessed how turnover influenced genetic differentiation among populations both temporally and spatially. We also examined the scale at which metapopulation dynamics occur in *A. uncatus*. We predicted that since mountains often act as barriers, dispersal occurs mostly within rather than between valleys, suggesting that extinction/re-colonisation dynamics occur more or less independently within valleys - that is, each valley may represent a metapopulation of its own.

## Methods

### Sampling scheme

**Figure 1:**
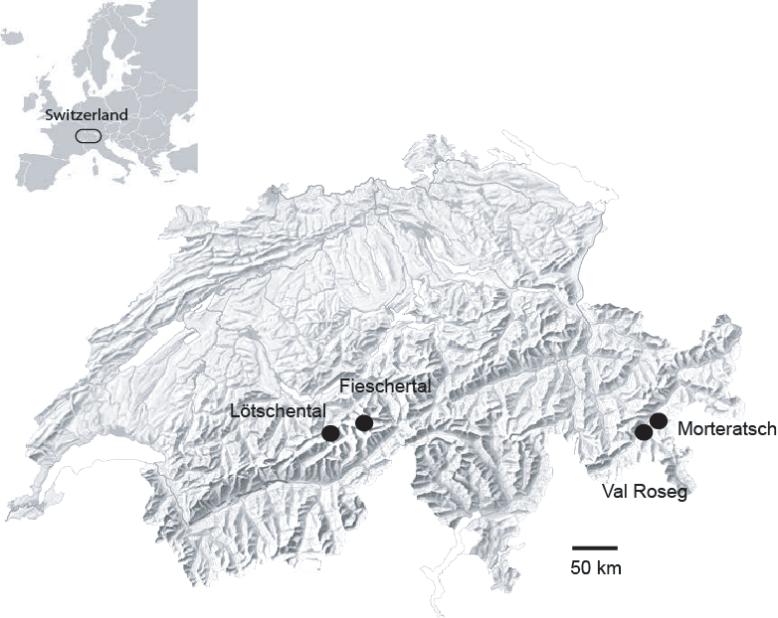
**Four valleys in the Swiss Alps where *A. uncatus *populations were sampled over multiple years**. Four valleys in the Swiss Alps where 8 *Allogamus uncatus *caddisfly populations were sampled before and after a major climate event, the European heat wave of 2003.

We sampled 8 caddisfly populations from different stream sites within each of four valleys in the Swiss Alps over multiple years (totalling 24 sampled populations; Figure 1, Table 1). Two adjacent valleys were sampled in both the eastern and western regions of the Swiss Alps: Engadin region (Morteratsch and Val Roseg) and Wallis region (Lötschental and Fieschertal). Within each valley, a permanent and a temporary stream population pair were sampled. None of the stream pairs were connected by stream flow at the sampling locations, thus larval dispersal (drift, crawling) between streams was unlikely, whereas adult dispersal (flying) between streams was possible. Populations were sampled prior to the climate event in 2003, and then in most cases, one year and five years later (in 2004 and 2008). In three populations (MUT, LLP, LLT), too few larvae were found in 2004 to warrant collection; populations crashed in 2003 and had not recovered by 2004. Consequently, these populations were sampled in 2005. In one population (MLP), only 8 larvae were collected in 2004 and no larvae were found in 2005, therefore, the sample size of MLP 2004 was n = 8. This population had recovered by 2008. The sample sizes of all other populations were 24-32 individuals (Table 1).

**Table 1:** Collection sites of *Allogamus uncatus *populations in the Swiss Alps and number of genotyped individuals.

Population code	Valley	Stream hydroperiod	Coordinates	2003	2004/5	2008
				n	n	n
MLP	Morteratsch	perm	N 46° 26' 26.8'' E 9° 56' 10.4'	24	8	24
MUT	Morteratsch	temp	N 46° 26' 11.9'' E 9° 56' 2.3''	24	24	24
VRLP	Val Roseg	perm	N 46° 25' 21.9" E 9° 51' 24.5"	32	24	24
VRST	Val Roseg	temp	N 46° 24' 52.1" E 9° 51' 15.4"	24	24	24
LLP	Lötschental	perm	N 46° 26' 33.2" E 7° 53' 37.0"	24	24	24
LLT	Lötschental	temp	N 46° 26' 34.0" E 7° 53' 39.0"	24	24	24
FLP	Fieschertal	perm	N 46° 27' 00.5" E 8° 07' 45.4"	24	24	24
FUT	Fieschertal	temp	N 46° 27' 08.1" E 8° 07' 37.8"	24	24	24

Collection sites with population code, valley, stream hydroperiod regime (perm = permanent; temp = temporary), coordinates, and the number of larvae sampled and genotyped (n) in different years.

### DNA extraction and microsatellite genotyping

Third or 4th instar larvae were collected from 10 - 100 m of stream length, depending on stream size. Larvae were either frozen at -80°C or stored in 96% ethanol for subsequent analysis. Genomic DNA was extracted from larval abdominal tissue (dried at 55°C for 1 h) using Nucleospin Plant^® ^DNA extraction kits (Macherey-Nagel). The seven microsatellite loci used in this study have been described elsewhere [30]. Four loci are di-nucleotide repeats (Allo 04, -09, -11, and -17), one locus is a tri-nucleotide repeat (Allo 22), one locus is a septa-nucleotide repeat (Allo 06), and one locus is a nine-nucleotide repeat (Allo 20). Loci were multiplexed in two groups: (1) Allo 04, Allo 06, Allo 17, Allo 20, and (2) Allo 09, Allo 11, Allo 22. Multiplex PCR reactions included 6 *μ*l of QIAGEN Multiplex Mastermix solution, 0.3 *μ*M of each primer, 5-10 ng DNA, 1.2 *μ*L QIAGEN Q solution, and ddH20 to make up a total reaction volume of 12 *μ*L. Forward primers were labelled with one of the fluorescent dyes G-FAM, VIC, NED or PET (DS-33, Applied Biosystems). PCR cycling conditions for both multiplex groups were: 15 min at 95°C, 30 cycles of 30 sec at 94°C, 2 min at 50°C, and 90 sec at 72°C, followed by 30 min at 60°C. Fragments were analysed on a 3130xl Genetic Analyzer using LIZ500 internal size standard and Genemapper v.4.7 software (Applied Biosystems).

### Genetic data analysis

Basic data on the genetic composition of populations (mean number of alleles, observed and expected heterozygosity) were computed using GENETIX [31]. F-statistics and tests for Hardy-Weinberg equilibrium and linkage disequilibrium were calculated using FSTAT v.2.9.3.2 [32]. Significant differences among sampling years in allelic richness (A*r*), observed heterozygosity (H_o_), unbiased gene diversity (H_s_) and F_ST _(averaged over the 8 populations) were tested using a permutation procedure (10,000 iterations) in FSTAT. Bonferroni corrections [33] were applied whenever multiple comparisons were made using the same procedure. Within each population, all loci were checked for the presence of null alleles using MICRO-CHECKER v.2.2.3 [34]. The overall frequency of null alleles (r) in each population was estimated using the method of Brookfield [35]. We also used the IIM approach (individual inbreeding model) in INEST [36] to partition out the influence of null alleles on F_IS _values. In addition, we estimated global and pairwise population F_ST _using FreeNA [37] to obtain unbiased F_ST _values in the presence of null alleles. Analysis of molecular variance (AMOVA) [38] was used to partition total molecular variance into that contributed by valley and stream hydroperiod regime using ARLEQUIN v.3.11 [39]. Finally, factorial correspondence analysis (FCA) based on allele frequencies implemented in GENETIX was used to illustrate the two-dimensional spatial representation of genetic differentiation among populations over multiple years.

We tested for signatures of genetic bottlenecks in all 24 sampled populations using two methods. First, heterozygosity excess was tested using BOTTLENECK v.1.2.02 [40]. We assumed a two-phase mutation model with 95% stepwise mutations, 5% multiple-step mutations, and a variance among multiple steps of 12 as recommended for microsatellites by Piry *et al. *[40]. Significance of heterozygosity excess over all loci (P) was determined with a one-tailed Wilcoxon sign rank test. Second, we used the *M*-ratio method developed by Garza & Williamson [41]. This approach calculates the ratio (*M*) of the total number of alleles to the range in allele sizes and *Mc*, the critical value of *M *(5% of values fall below *Mc *as determined by simulations). *M *and *Mc *were estimated using M_P_VAL and CRITICAL_M, respectively [41]. Both programs require three input parameters: (1) θ, which = 4N_e_μ, (2) p_g_, the percent mutations larger than single step, and (3) Δg, mean size of mutations larger than single step. We set the average mutation rate to 5 × 10^-4^, p_g _to 0.10 and Δg to 3.5 as recommended by Garza & Williamson [41], and each set of simulations consisted of 10,000 iterations. Estimating N_e _for local populations within a metapopulation setting is problematic and potentially biased, as most methods assume a closed population and do not account for immigration, thus often underestimating the true N_e _[42]. Moreover, at least one method currently available to estimate N_e _and *m *(number of migrants) simultaneously [43] requires *apriori *knowledge or assumptions to be made about potential source populations. Therefore, we estimated N_e _using two standard methods to get a range of plausible population-specific values, but also set N_e _to values above this range (thereby increasing θ) for a more conservative *M*-ratio test [see also [44,45]]. We estimated population-specific N_e _using the linkage disequilibrium method and a temporal method [46] in N_e_ESTIMATOR v.1.3 [47], and tested three conservative values of θ that equate to N_e _of 100, 500 and 1000.

We estimated gene flow (m = proportion of migrants) between population pairs using BayesASS+ v.1.3 [48]. We chose to use BayesASS+ because it estimates recent gene flow (i.e. migration rates over the last several generations) using MCMC techniques and does not assume that populations are in migration-drift balance or Hardy-Weinberg equilibrium [see also [49]]. We ran the program separately for each sampling year. Initial runs were performed to determine delta values for allele frequency, migration rate, and inbreeding that ensured that proposed changes between chains at the end of the run were between 40-60% of the total chain length as recommended by Wilson & Rannala [48]. Samples were collected every 2000 iterations to infer posterior probability distributions of migration rates. We then ran 2 subsequent runs for each sampling year using 6 × 10^6 ^iterations and different random number seeds to confirm chain convergence. Migration rates and 95% confidence intervals were determined for each population pair.

## Results

### Genetic diversity within populations

The seven microsatellite loci surveyed were highly polymorphic; a total of 126 different alleles (mean = 18.0) were found in the 24 *A. uncatus *sampled populations (Table 2). Mean number of alleles ranged from 6.71 - 9.00 in 2003 populations, from 4.86 - 8.00 in 2004/5, and from 5.14 - 7.43 in 2008. Allelic richness and gene diversity (averaged over loci and populations in each year) decreased significantly over time (FSTAT comparison among groups: p = 0.001 and p = 0.020, respectively), whereas observed heterozygosity showed no significant increase or decrease among years (p = 0.10; Table 2). No significant linkage disequilibrium was detected in 252 pairwise tests between loci.

**Table 2:** Population-specific microsatellite diversity and frequency of null alleles for *A. uncatus *populations sampled over multiple years.

			2003					2004/5					2008		
Popn	A	H_o_	H_e_	F_IS_	r	A	H_o_	H_e_	F_IS_	r	A	H_o_	H_e_	F_IS_	r
MLP	7.57	0.534	0.707	0.268*	0.101^†^	4.86	0.607	0.632	0.105	0.015	6.29	0.607	0.635	0.066	0.017
MUT	9.00	0.489	0.737	0.359*	0.143^†^	6.57	0.631	0.635	0.028	0.003	6.57	0.696	0.685	0.004	-0.007
VRLP	7.43	0.401	0.709	0.452*	0.180^†^	5.43	0.485	0.557	0.152	0.046^†^	5.57	0.564	0.556	0.007	-0.005
VRST	6.57	0.491	0.644	0.260*	0.093^†^	5.29	0.542	0.589	0.103	0.029^†^	5.14	0.524	0.554	0.076	0.019
LLP	8.57	0.608	0.795	0.251*	0.104^†^	7.14	0.589	0.676	0.148	0.051^†^	6.14	0.503	0.627	0.222*	0.077^†^
LLT	9.00	0.512	0.823	0.399*	0.171^†^	6.71	0.598	0.679	0.142	0.049^†^	5.71	0.521	0.590	0.140	0.044^†^
FLP	6.71	0.499	0.739	0.353*	0.138^†^	7.86	0.691	0.782	0.138*	0.051^†^	7.71	0.641	0.727	0.150	0.049^†^
FUT	8.14	0.647	0.786	0.194*	0.078^†^	8.00	0.606	0.775	0.241*	0.095^†^	7.43	0.577	0.718	0.224*	0.082^†^

mean	5.63	0.525	0.766	0.315	0.126	4.69	0.596	0.686	0.131	0.042	4.41	0.592	0.653	0.093	0.035

Population codes (Popn) as in Table 1, mean number of alleles (A), observed and expected heterozygosity (H_o _and H_e_), deviations from HWE estimated as F_IS _in FSTAT (*denotes significance after Bonferroni correction), and estimated frequency of null alleles (r) using the method of Brookfield [35], where † denotes null alleles detected in at least one locus using MICROCHECKER [34]. Means averaged over loci and populations for each sampling year.

We found a significant deviation from Hardy-Weinberg equilibrium (HWE) in all 8 populations in 2003, as well as in two populations in both 2004/5 and in 2008 (Table 2). Null alleles were detected in at least one locus for 18/24 samples. The overall frequency of null alleles (r) in each population ranged from 0.180 to zero. The mean frequency of null alleles declined from 0.126 in 2003 to 0.035 in 2008 (Table 2). Locus Allo 17 showed null alleles in all 12 Wallis region samples, as well as in three Engadin populations sampled in 2003 (MUT, MLP, VRLP). Excluding this locus from the analysis resulted in fewer samples out of HWE (7 instead of 12). The pattern of decreasing allelic richness and gene diversity over time, and no change in observed heterozygosity, remained significant when Allo 17 was excluded from the analysis (p = 0.017, 0.049 and 0.236, respectively). Subsequent analyses (e.g. bottleneck detection, N_e _and migration rate estimation) were performed with and without Allo 17 for comparison (see below). Deviations from HWE were primarily due to the presence of null alleles and not inbreeding since F_IS _values were reduced to essentially zero in all 24 sampled populations when null alleles were accounted for (IIM F_IS _range: 0.012 - 0.063). It is important to note that although the frequency of null alleles was fairly high in some 2003 samples, several lines of evidence suggest that any potential bias stemming from these is not likely to alter the outcome of our study. For instance, simulation studies have shown that null alleles lead to an underestimation of allelic diversity and observed and expected heterozygosity, but that this bias is particularly low for expected heterozygosity [50]. Any underestimation of genetic diversity within 2003 samples would actually dampen the pattern of decline in these parameters after 2003. Moreover, we did not find a significant increase in observed heterozygosity after 2003.

### Genetic differentiation among populations

**Figure 2:**
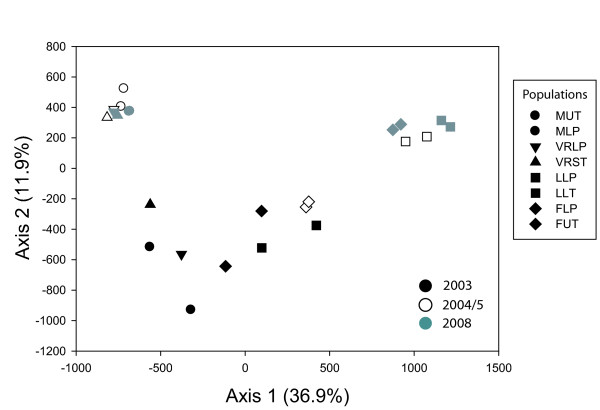
**Factorial correspondence analysis depicting genetic differentiation among *A. uncatus *populations over multiple years**. Factorial correspondence analysis based on allele frequencies of *A. uncatus *depicting the spatial representation of genetic differentiation among populations over multiple years. Inertia of each axis is given in parentheses. Population codes as in Table 1.

Genetic differentiation among valleys increased significantly after 2003, whereas differentiation between populations within a given valley remained low (Additional file 1 - Pairwise F_ST _values for the 8 *A. uncatus *populations sampled over multiple years, Figure 2). F_ST _(averaged over populations in each sampling year) increased from 0.058 in 2003 to 0.166 in 2004/5 and 0.209 in 2008 (p = 0.001). Global F_ST _(over all 24 sampled populations) estimated from uncorrected genotypes was 0.152 (CI: 0.129 - 0.179) and 0.148 (CI: 0.126 - 0.174) when using the ENA method in FreeNA [37], indicating that null alleles had only a small effect on F_ST _estimates. Single locus F_ST _values as well as pairwise F_ST _estimates also were very similar with or without correction for null alleles (corrected data not shown). AMOVA revealed that valley comprised a significant proportion of the total molecular variance whereas stream hydroperiod regime did not (Table 3). These results also were reflected in analyses of pairwise population differentiation (F_ST_; Additional file 1) and factorial correspondence analysis (Figure 2). Populations sampled within a valley in a given year did not differ significantly from each other - that is, permanent and temporary streams did not differ (F_ST_; Additional file 1), and stream hydroperiod regime accounted for little of the total genetic variance in each sampling year (Table 3). Valleys differed significantly from each other in 2004/5 and 2008, but not in 2003 (Table 3). In 2003, pairwise F_ST _values between regions ranged from 0.018 to 0.148, and were similar to F_ST _values within regions (0.014 to 0.089). By 2008, pairwise F_ST _values between regions ranged from 0.226 to 0.390, whereas F_ST _values within regions remained low and ranged from 0.022 to 0.073 (F_ST_; Additional file 1). In concordance, the FCA illustrates that after 2003, populations in the Engadin region were strongly differentiated from populations in the Wallis region (Figure 2).

**Table 3:** Analysis of molecular variance (AMOVA) for the 8 *A. uncatus *populations in each sampling year.

Source of variation	**d.f**.	% of variation	P-value	ϕ - statistics
**2003**				
Among valleys	3	2.64	0.057	0.052
Populations within valleys	4	2.55	< 0.001	0.026
Within populations	392	94.81	< 0.001	0.026
**2004/2005**				
Among valleys	3	17.90	0.005	0.187
Populations within valleys	4	0.76	0.061	0.009
Within populations	344	81.34	< 0.001	0.179
**2008**				
Among valleys	3	22.04	0.006	0.222
Populations within valleys	4	0.19	0.361	0.002
Within populations	376	77.77	< 0.001	0.220

d.f. = degrees of freedom

### Genetic bottlenecks

Bottlenecks were detected in all 8 populations (both permanent and temporary streams) at some point during 2003 - 2008 using the *M*-ratio method (Table 4), but in only one population using the heterozygosity excess method. Only VRLP 2003 had a significant probability of heterozygosity excess (P = 0.020) based on the Wilcoxon sign rank test implemented in BOTTLENECK (all other populations P > 0.148). The results were qualitatively the same when locus Allo 17 was excluded (VRLP 2003 P = 0.023; all other populations P > 0.281), providing support for the tenet that null alleles are a minor source of error in estimating heterozygosity excess for the detection of bottlenecks [13]. In contrast to the first method, *M*-ratio analyses revealed signatures of genetic bottlenecks in all 8 populations in 2003, 2004/2005, and in 7/8 populations in 2008. These results also were the same with or without Allo 17 (results without Allo 17 are shown). Observed *M*-ratios ranged between 0.542 - 0.879 and were lower than the corresponding critical *M *in all but one population (LLP 2008) when θ was calculated using both population-specific N_e _estimates (Table 4). Population-specific N_e _estimates ranged from 8.5 - 559.5 using the linkage disequilibrium method (two populations were estimated as ∞ likely due to model nonconvergence), and from 3.9 - 33.0 using the temporal method. There was a tendency for N_e _to increase after 2003, but no clear pattern of change was seen after 2004/2005 (Table 4). Our conservative tests revealed the same pattern as population-specific tests when N_e _= 100 was assumed (except for VRLP 2003), but not when N_e _was assumed to be 500 or 1000. When N_e _was set to 500 (θ = 1), signatures of genetic bottlenecks were no longer detectable in VRLP 2003, FUT 2003, MUT 2005, and three populations in 2008 (MUT, LLP and LLT). When N_e _was set to 1000, bottlenecks were detected in 10/24 populations (Table 4).

**Table 4:** N_e _and *M*-ratio analyses of *A. uncatus *populations sampled before/after the European heat wave of 2003.

			2003				2004/5				2008	
Popn	N_e _LD	N_e _temp	*M*	*Mc*	N_e _LD	N_e _temp	*M*	*Mc*	N_e _LD	N_e _temp	*M*	*Mc*
MLP	17.9	6.5	0.712	*/*/*/*/ns	34.4	33.0	0.542	*/*/*/*/*	43.0	11.5	0.736	*/*/*/*/ns
MUT	41.5	4.2	0.738	*/*/*/*/ns	53.7	17.7	0.750	*/*/*/ns/ns	559.5	8.7	0.759	*/*/*/ns/ns
VRLP	42.7	3.9	0.815	*/*/ns/ns/ns	39.9	10.0	0.657	*/*/*/*/*	70.6	7.7	0.738	*/*/*/*/ns
VRST	13.1	4.0	0.732	*/*/*/*/ns	33.2	10.8	0.732	*/*/*/*/ns	∞	8.2	0.600	*/*/*/*/*
LLP	55.5	5.4	0.664	*/*/*/*/*	257.5	9.1	0.674	*/*/*/*/*	94.2	9.9	0.879	ns/ns/ns/ns/ns
LLT	44.4	4.2	0.739	*/*/*/*/ns	∞	7.7	0.692	*/*/*/*/*	68.3	6.2	0.764	*/*/*/ns/ns
FLP	8.5	8.0	0.615	*/*/*/*/*	68.9	6.1	0.741	*/*/*/*/ns	45.0	6.1	0.633	*/*/*/*/*
FUT	25.7	14.3	0.750	*/*/*/ns/ns	360.0	4.4	0.682	*/*/*/*/*	104.5	8.1	0.680	*/*/*/*/*

Population codes (Popn) as in Table 1. Ne estimated using the linkage disequilibrium (LD) and temporal method (temp) in NeESTIMATOR [47]. Where N_e _was estimated as ∞, N_e _LD = 500 was used for *M*. *M *was averaged over 6 loci in each sample. *Mc *values (critical *M*) were generated using five values of θ: two population-specific values based on N_e_LD and N_e_temp, and three general values using N_e _of 100, 500 and 1000. * denotes significance of *M *tested against *Mc *(*M *lower than *Mc*) at p = 0.05. ns = nonsignificant.

Estimates of migration rates were significantly asymmetric (non-overlapping 95% C.I.) for 4 population pairs in 2003, 5 population pairs in 2004/2005 and 4 population pairs in 2008. Migration rates ranged from 0.002 to 0.288 and were qualitatively the same with and without Allo 17 (without Allo 17 shown; Additional file 2 - Directional gene flow estimates (m) for each pair of *A. uncatus *populations sampled over multiple years). In 2003, across-valley migrants were identified within each region: VRLP was a net exporter of migrants into MLP and MUT, and FLP was a net exporter of migrants into LLP and LLT (Additional File 2 - Directional gene flow estimates, Table 5). In 2004/5, across-valley migrants were identified in the Engadin region but not in the Wallis region: VRLP was a net exporter of migrants into MLP, MUT and VRST, LLP was a net exporter of migrants into LLT, and FLP was a net exporter of migrants into FUT. In 2008, within each of the 4 valleys, the permanent stream was a net exporter of migrants into the temporary stream (e.g. MLP into MUT), but no across-valley migrants were identified (Additional File 2 - Directional gene flow estimates, Table 5).

**Table 5:** Emigration (E), immigration (I) and net emigration (net E) rates among *A. uncatus *populations sampled over multiple years.

		2003			2004/5			2008	
Popn	E	I	net E	E	I	net E	E	I	net E
									
MLP	0.037	0.320	-0.284	0.030	0.301	-0.272	0.259	0.022	0.237
MUT	0.039	0.314	-0.276	0.044	0.320	-0.276	0.026	0.321	-0.295
VRLP	0.659	0.015	0.644	0.794	0.014	0.780	0.366	0.020	0.346
VRST	0.109	0.021	0.087	0.041	0.320	-0.280	0.028	0.321	-0.293
LLP	0.163	0.208	-0.044	0.330	0.012	0.318	0.333	0.015	0.318
LLT	0.035	0.318	-0.283	0.040	0.320	-0.280	0.027	0.321	-0.294
FLP	0.473	0.037	0.435	0.307	0.038	0.269	0.299	0.027	0.272
FUT	0.037	0.317	-0.281	0.045	0.305	-0.260	0.029	0.320	-0.292

Population codes (Popn) as in Table 1. Total emigration (E) and total immigration (I) represent the sums of all pairwise gene flow estimates for each population (see Additional file 2 for directional gene flow estimates for each population pair). Net emigration rates represent the sum of all emigration rates minus the sum of all immigration rates for each population.

## Discussion

Metapopulations are characterised by interconnected subpopulations with local extinction and re-colonisation turnover dynamics [1,2]. Population turnover should lead to a loss of genetic variation, and populations may become more differentiated due to bottlenecks during colonisation and the action of genetic drift [16]. Our results are consistent with these predictions. Here, we detected reductions in genetic diversity and signatures of genetic bottlenecks after an extreme climate event in all of our tested populations. Our results further show that local population turnover led to large changes in allele frequencies among valleys within one to two years (1-2 generations), most likely attributable to genetic drift and immigration. Consequently, patterns of genetic differentiation among *A. uncatus *populations changed markedly both temporally and spatially in response to environmental change.

### Genetic consequences of population turnover

Several types of genetic changes are associated with population turnover, such as loss of alleles, transient heterozygosity excess, and changes in the distribution of allele frequencies [9]. We found that both permanent and temporary stream populations from all four valleys underwent significant turnover by 2008 relative to 2003, reflecting the impact of the 2003 climate event on the populations. Consistent with theory, populations showed a decline in allelic richness and gene diversity, and experienced large changes in allele frequencies after 2003. Similar patterns of genetic change after bottlenecks have recently been shown for some vertebrate taxa [e.g. [45,51]], and highlight the importance of sufficient genetic diversity to cope with bottleneck events and to ultimately maintain population persistence in the long term. Like several other studies, we did not detect a transient excess of heterozygosity after turnover [see also [44]]. The lack of heterozygosity excess after a bottleneck is most often attributed to (i) the sensitivity of the detection methods, and (ii) population recovery (expansion and immigration). We discuss both of these potential explanations in more detail below.

### Signatures of genetic bottlenecks

Bottlenecks were detected in all 8 populations after 2003 using the *M*-ratio method, but in no populations when using a heterozygosity excess method, indicating differential sensitivity of bottleneck detection methods. The pattern of genetic changes after population turnover found in our study is consistent with the theoretical expectation that allelic diversity is more sensitive to bottlenecks than heterozygosity, at least in the short term [11]. Consequently, methods based on measures of heterozygosity (e.g. BOTTLENECK) are expected to be less sensitive, as demonstrated in several recent empirical studies [44,45,51]. Moreover, strong bottleneck signatures may not be detected by this method in populations that fluctuate in size where N_e _is initially low and reductions in census size do not generate strong reductions in N_e _[13], as is likely to be the case in our tested populations. In contrast, *M*-ratios appear to be more sensitive in detecting bottlenecks and are more robust to violations of model assumptions such as lack of mutation-drift equilibrium, admixture and population subdivision *i.e. *metapopulation structure [45]. Interestingly, *M*-ratio analyses also detected signatures of bottlenecks in all 2003 samples, indicating that populations experienced at least one bottleneck prior to the climate event of 2003. This is not surprising for populations inhabiting ephemeral habitats, or for high altitude populations that face extreme environmental conditions as these often do experience fluctuations in population size [45,52].

Bottlenecks are usually accompanied by reductions in N_e _[11]; however, we found a trend of increased N_e _after 2003, suggesting a strong influence of immigration in maintaining or even increasing population size after bottleneck events [13]. Admittedly, our population-specific N_e _estimates may suffer from several sources of potential bias (null alleles, open population, metapopulation setting). However, the focus of our study was not N_e_, but rather, whether bottlenecks occurred after 2003 and the consequent genetic changes in our study populations. Although we did not quantify census population size, our best guess based on field observations would range from 50-500 (depending on stream size), and with N_e _lower than this. Given this range, results from our conservative tests based on N_e _= 100 and 500 are most plausible, with the true N_e _likely somewhere in between. For both N_e _estimates, signatures of genetic bottlenecks were detected in all or nearly all (7/8) populations after the climate event and observed demographic crash of 2003.

Our estimates of directional gene flow revealed that migration between populations both within and among valleys plays an important role in maintaining population size and genetic diversity after bottleneck events. Prior to 2003, across-valley migrants were identified in both regions, suggesting that genetically differentiated valleys occasionally exchange migrants that contribute to the maintenance of local genetic diversity [14]. After the climate event of 2003, in the Engadin region, VRLP was not only a source of within-valley migrants to a temporary stream (VRST), but also a likely source of new alleles for streams in an adjacent valley. In contrast, we did not detect across-valley migrants in populations of both valleys of the Wallis region after 2003, suggesting that re-colonisation patterns were more consistent with local expansion of different remnant demes [see also [14]]. Of course, the detection of migrants is strongly dependent on our sampling scheme, as all potential sources of immigrants were not sampled. By 2008, no across-valley migrants were identified, but within each valley, permanent streams were a source of local migrants to temporary streams. In other words, each valley was maintaining its local genetic diversity as a source-sink metapopulation [2].

### Population turnover, genetic differentiation and genetic structure

Whether turnover will increase or decrease genetic differentiation among populations depends on the mode of colonisation [5]. We found that within valleys, permanent and temporary stream populations were not genetically differentiated, and this result was consistent over multiple years. This pattern suggests either that permanent stream individuals colonise temporary streams after local extinction, or that in extreme years (like 2003) when streams of both hydroperiod regimes experience local extinction, both are colonised by a random sample of individuals from the metapopulation as a whole [10]. In both scenarios, ongoing gene flow and admixture homogenize allele frequencies among populations within a valley [4,53]. Our results are in accordance with those of a separate study where we investigated gene flow among 6 populations within one valley (Val Roseg), and found high levels of admixture and low population differentiation [54].

Turnover is expected to increase genetic differentiation among populations when colonisers into each population originate from differentiated sources (propagule-pool model) [10]. A similar pattern of increased differentiation among populations can arise if re-colonisation is dominated by the offspring of very few individuals (patchy recruitment hypothesis), which has been shown in several studies of aquatic insects [e.g. [55]]. We found that among valley differentiation increased markedly after turnover, and underlying changes in allele frequencies were consistent with aspects of both of these models. More specifically, overall genetic differentiation was significantly higher in 2008 relative to 2003, and turnover within all four valleys increased genetic differentiation among valleys and changed the apparent spatial structure of populations over time. Several studies have documented temporal changes in genetic differentiation among populations [19-21], and some in the context of bottleneck events [44,45], but very few have shown concomitant changes in spatial structure [but see [14]]. The pattern of spatio-temporal genetic changes found in our study can be compared to that of cyclic populations, where demographic crashes lead to a patchy population structure with local, isolated demes of small size experiencing strong effects of drift. During population recovery, gene flow among these genetically differentiated demes leads to lower overall differentiation [14]. Here, the lack of spatial genetic structure among populations prior to but not after turnover in 2003 is consistent with the above scenario, but it remains to be tested when (or if) future gene flow among valleys leads to subsequent homogenisation.

Our study provides a rare example of how monitoring temporal and spatial changes in genetic differentiation after bottlenecks allows us to assess how the competing processes of drift and immigration act on the genetic structure of populations [see also [14,20]]. Although high values of genetic differentiation measures (such as F_ST_) are conventionally interpreted as indicating a long period of time since divergence [11], these measures are not proportional to divergence time when a bottleneck has occurred [56]. After a recent bottleneck, high F_ST _values between pre- and post-bottlenecked samples are usually interpreted as an indication of rapid genetic drift and increased among population variance due to loss of different alleles in different populations [4,5,16,57]. Alternatively, the arrival of new alleles after a bottleneck event via immigration also can drive increased F_ST _among populations when immigrants originate from differentiated sources [14,44]. Indeed, the loss of rare alleles due to drift can be balanced by rapid accumulation of new alleles from immigration, the result being that relatively high genetic diversity can be maintained in populations that fluctuate in size [14]. Both drift and immigration are likely contributing to the changes in genetic structure seen in our studied populations as reflected by the observed patterns in F_ST_, the identification of across-valley migrants, and the lack of reductions in N_e _after the bottleneck event.

### Metapopulations of *A. uncatus*

In this study, we found that turnover of local populations of *A. uncatus *led to high levels of admixture within valleys, but greater differentiation among valleys. Based on these results, we suggest that valleys represent 'semi-independent networks' *sensu *Hanski [2] and are the most likely units of metapopulations for this species. Yet, prior to population turnover (2003), regions were not strongly differentiated indicating that gene flow between valleys over time could erode the effects of founding events quite quickly [18]. Models of future climate change predict not only an increase in average temperatures, but also an increase in extreme weather events such as the European heat wave of 2003 [29]. Under this scenario, we predict that the frequency of population turnover events also will increase. If this increase in frequency occurs, it is possible that genetic differentiation between valleys may not break down completely before the next event, leading to a more insular structure with valleys always being differentiated. Increased insularity of valleys could lead to reduced genetic variation of local populations due to a lack of immigration from adjacent valleys, and may have long-term detrimental effects on *A. uncatus *populations' abilities to cope with future environmental change.

## Conclusions

We conclude that the European heat wave in 2003 was associated with bottlenecks that led to reduced genetic diversity within all of our studied populations. Local population turnover led to large changes in allele frequencies among valleys over time, driven by genetic drift and immigration. Our results highlight the magnitude of temporal and spatial population genetic changes in response to extreme climate events, and demonstrate how such events can cause rapid genetic divergence in metapopulations.

## Authors' contributions

LNSS, CTR and JJ conceived and designed the study. LNSS, CTR and KBK conducted field sampling. LNSS and KBK acquired and analysed the data. All authors were involved with interpreting the data. LNSS drafted the manuscript. KBK, CTR and JJ provided critical revisions. All authors have approved the final version.

## Supplementary Material

Additional file 1 **Pairwise F_ST _values for the 8 *A. uncatus *populations sampled over multiple years**. F_ST _values are shown below and significance is shown above the diagonal. Population codes as in Table 1. * indicates significance after Bonferroni correction, NS = nonsignificant. Click here for file

Additional file 2 **Directional gene flow estimates (m) for each pair of *A. uncatus *populations sampled over multiple years**. Gene flow estimates represent the mean migration rate and 95% confidence intervals (in parentheses) for a pair of populations. Click here for file
