# Supplementary material for: Bottlenecks drive temporal and spatial genetic changes in alpine caddisfly metapopulations
Source: BMC Evol Biol. 2011 Sep 27;11:278. doi: 10.1186/1471-2148-11-278 (PMC3188517; doi:10.1186/1471-2148-11-278)
Supplement: Additional file 1 — Pairwise FST values for the 8 A. uncatus populations sampled over multiple years. FST values are shown below and significance is shown above the diagonal. Population codes as in Table 1. * indicates significance after Bonferroni correction, NS = nonsignificant. [file 1471-2148-11-278-S1.PDF]

Additional file 1 - Pairwise F_{ST} values for the 8 *A. uncatus* populations sampled over multiple years.

	MUT03	MLP03	MUT05	MLP04	MUT08	MLP08	VRLP03	VRST03	VRLP04	VRST04	VRLP08	VRST08	LLP03	LLT03	LLP05	LLT05	LLP08	LLT08	FLP03	FUT03	FLP04	FUT04	FLP08	FUT08
MUT03		NS	*	NS	*	*	NS	NS	*	*	*	*	*	NS	*	*	*	*	NS	*	*	*	*	NS
MLP03	0.022		*	*	*	*	*	*	*	*	*	*	*	*	*	*	*	*	NS	*	*	*	*	*
MUT05	0.135	0.085		NS	*	*	*	*	*	*	*	*	*	*	*	*	*	*	NS	*	*	*	*	*
MLP04	0.132	0.073	0.001		NS	NS	NS	NS	NS	NS	*	*	*	*	*	*	*	*	NS	*	*	*	*	NS
MUT08	0.115	0.076	0.012	0.004		NS	*	*	*	*	*	*	*	*	*	*	*	*	*	*	*	*	*	*
MLP08	0.137	0.084	0.025	0.005	-0.001		*	*	*	*	*	*	*	*	*	*	*	*	NS	*	*	*	*	*
VRLP03	0.014	0.033	0.103	0.099	0.086	0.104		NS	*	*	*	*	*	NS	*	*	*	*	NS	NS	*	*	NS	*
VRST03	0.089	0.085	0.068	0.086	0.061	0.072	0.062		*	*	*	*	*	*	*	*	*	*	NS	*	*	*	NS	*
VRLP04	0.160	0.108	0.016	0.035	0.036	0.034	0.116	0.090		NS	*	*	*	*	*	*	*	*	NS	*	*	*	*	*
VRST04	0.149	0.095	0.012	0.012	0.018	0.020	0.104	0.084	-0.004		NS	*	*	*	*	*	*	*	NS	*	*	*	*	*
VRLP08	0.171	0.127	0.039	0.077	0.032	0.042	0.129	0.107	0.037	0.024		NS	*	*	*	*	*	*	NS	*	*	*	*	*
VRST08	0.169	0.128	0.040	0.062	0.028	0.022	0.117	0.093	0.031	0.020	0.017		*	*	*	*	*	*	NS	*	*	*	*	*
LLP03	0.087	0.118	0.156	0.129	0.129	0.152	0.092	0.148	0.197	0.180	0.196	0.190		NS	*	*	*	*	NS	NS	*	NS	*	*
LLT03	0.037	0.056	0.107	0.082	0.086	0.111	0.052	0.091	0.144	0.127	0.152	0.152	0.004		*	*	*	*	NS	NS	NS	NS	*	*
LLP05	0.219	0.253	0.277	0.248	0.248	0.270	0.219	0.279	0.322	0.304	0.328	0.319	0.068	0.112		NS	*	*	NS	*	*	*	*	*
LLT05	0.212	0.247	0.257	0.238	0.228	0.252	0.211	0.254	0.305	0.288	0.304	0.299	0.050	0.094	0.019		*	*	NS	*	*	*	NS	*
LLP08	0.254	0.282	0.317	0.287	0.283	0.304	0.251	0.318	0.362	0.343	0.365	0.358	0.078	0.138	0.040	0.038		NS	NS	*	*	*	NS	*
LLT08	0.275	0.305	0.342	0.321	0.312	0.330	0.278	0.341	0.387	0.369	0.390	0.384	0.099	0.150	0.057	0.049	-0.004		NS	*	*	*	NS	*
FLP03	0.022	0.057	0.108	0.105	0.094	0.118	0.018	0.079	0.134	0.116	0.130	0.130	0.048	0.015	0.174	0.153	0.206	0.226		NS	NS	NS	NS	NS
FUT03	0.049	0.075	0.116	0.114	0.088	0.115	0.055	0.082	0.145	0.132	0.143	0.132	0.036	0.014	0.140	0.114	0.174	0.188	0.023		NS	NS	NS	NS
FLP04	0.088	0.124	0.157	0.144	0.133	0.160	0.100	0.140	0.195	0.179	0.194	0.194	0.014	0.018	0.102	0.075	0.132	0.147	0.039	0.006		NS	*	*
FUT04	0.102	0.124	0.170	0.152	0.139	0.163	0.111	0.160	0.207	0.184	0.198	0.192	0.026	0.024	0.120	0.100	0.123	0.132	0.060	0.016	0.016		*	*
FLP08	0.189	0.215	0.255	0.222	0.226	0.249	0.195	0.250	0.306	0.289	0.304	0.301	0.066	0.090	0.055	0.049	0.073	0.073	0.146	0.091	0.058	0.085		NS
FUT08	0.200	0.225	0.259	0.224	0.234	0.256	0.206	0.261	0.306	0.292	0.307	0.301	0.069	0.101	0.059	0.060	0.073	0.069	0.153	0.104	0.074	0.084	-0.010	

color legend: streams within a valley; population turnover; no change

F_{ST} values are shown below and significance is shown above the diagonal. Population codes as in Table 1. * indicates significance after Bonferroni correction, NS = nonsignificant.
