# Supplementary material for: Bottlenecks drive temporal and spatial genetic changes in alpine caddisfly metapopulations
Source: BMC Evol Biol. 2011 Sep 27;11:278. doi: 10.1186/1471-2148-11-278 (PMC3188517; doi:10.1186/1471-2148-11-278)
Supplement: Additional file 2 — Directional gene flow estimates (m) for each pair of A. uncatus populations sampled over multiple years. Gene flow estimates represent the mean migration rate and 95% confidence intervals (in parentheses) for a pair of populations. [file 1471-2148-11-278-S2.PDF]

Additional file 2 - Directional gene flow estimates (m) for each pair of *A. uncatus* populations sampled over multiple years.

| | Gene flow (m) | | |
|---|---|---|---|
| Population pair | 2003 | 2004/2005 | 2008 |
| MLP to MUT | 0.007 ($4.6e^{-06}$-0.035) | 0.005 ($5.3e^{-07}$-0.030) | 0.227 (0.125-0.307) |
| MLP to VRLP | 0.002 ($9.8e^{-11}$-0.015) | 0.002 ($3.9e^{-11}$-0.014) | 0.005 ($1.7e^{-10}$-0.039) |
| MLP to VRST | 0.003 ($8.0e^{-11}$-0.019) | 0.005 ($3.4e^{-07}$-0.033) | 0.011 ($1.0e^{-06}$-0.058) |
| MLP to LLP | 0.006 ($5.4e^{-07}$-0.033) | 0.002 ($9.3e^{-12}$-0.016) | 0.002 ($4.5e^{-11}$-0.015) |
| MLP to LLT | 0.008 ($6.8e^{-05}$-0.039) | 0.005 ($9.7e^{-07}$-0.028) | 0.005 ($1.2e^{-06}$-0.032) |
| MLP to FLP | 0.003 ($1.1e^{-09}$-0.026) | 0.004 ($3.9e^{-09}$-0.026) | 0.003 ($1.8e^{-10}$-0.021) |
| MLP to FUT | 0.009 ($9.3e^{-05}$-0.040) | 0.006 ($9.1e^{-07}$-0.035) | 0.006 ($1.2e^{-06}$-0.029) |
| MUT to MLP | 0.006 ($9.7e^{-07}$-0.033) | 0.017 ($7.8e^{-06}$-0.080) | 0.002 ($3.8e^{-11}$-0.019) |
| MUT to VRLP | 0.002 ($1.5e^{-10}$-0.013) | 0.002 ($8.9e^{-12}$-0.017) | 0.002 ($2.9e^{-11}$-0.018) |
| MUT to VRST | 0.002 ($8.2e^{-11}$-0.018) | 0.006 ($8.7e^{-07}$-0.032) | 0.005 ($4.0e^{-07}$-0.030) |
| MUT to LLP | 0.006 ($5.0e^{-07}$-0.039) | 0.002 ($4.3e^{-12}$-0.015) | 0.002 ($3.3e^{-11}$-0.014) |
| MUT to LLT | 0.009 ($7.6e^{-05}$-0.040) | 0.005 ($2.8e^{-07}$-0.027) | 0.005 ($6.3e^{-07}$-0.031) |
| MUT to FLP | 0.003 ($1.1e^{-09}$-0.022) | 0.007 ($1.8e^{-08}$-0.040) | 0.003 ($7.7e^{-10}$-0.020) |
| MUT to FUT | 0.010 (0.001-0.046) | 0.006 ($6.3e^{-07}$-0.037) | 0.006 ($1.7e^{-06}$-0.034) |
| VRLP to MLP | 0.283 (0.213-0.325) | 0.202 (0.081-0.311) | 0.008 ($4.8e^{-10}$-0.064) |
| VRLP to MUT | 0.254 (0.163-0.319) | 0.288(0.230-0.326) | 0.061 (0.005-0.157) |
| VRLP to VRST | 0.006 ($9.5e^{-11}$-0.042) | 0.288 (0.226-0.324) | 0.281 (0.214-0.323) |
| VRLP to LLP | 0.007 ($1.2e^{-06}$-0.039) | 0.002 ($6.0e^{-11}$-0.015) | 0.002 ($2.5e^{-11}$-0.018) |
| VRLP to LLT | 0.041 (0.001-0.103) | 0.005 ($6.2e^{-07}$-0.031) | 0.006 ($9.6e^{-07}$-0.034) |
| VRLP to FLP | 0.013 ($3.5e^{-10}$-0.069) | 0.004 ($6.2e^{-09}$-0.024) | 0.003 ($1.7e^{-09}$-0.019) |
| VRLP to FUT | 0.055 (0.002-0.153) | 0.005 ($6.1e^{-07}$-0.027) | 0.005 ($7.0e^{-07}$-0.029) |
| VRST to MLP | 0.007 ($1.4e^{-06}$-0.044) | 0.016 ($4.8e^{-06}$-0.079) | 0.003 ($9.2e^{-11}$-0.019) |
| VRST to MUT | 0.012 ($7.9e^{-06}$-0.062) | 0.005 ($9.8e^{-07}$-0.029) | 0.007 ($8.7e^{-06}$-0.033) |
| VRST to VRLP | 0.003 ($1.3e^{-10}$-0.022) | 0.002 ($1.2e^{-11}$-0.016) | 0.002 ($7.7e^{-11}$-0.021) |
| VRST to LLP | 0.008 ($6.0e^{-07}$-0.044) | 0.002 ($8.4e^{-12}$-0.017) | 0.002 ($2.1e^{-11}$-0.015) |
| VRST to LLT | 0.012 ($8.4e^{-05}$-0.053) | 0.005 ($1.3e^{-06}$-0.027) | 0.006 ($1.0e^{-06}$-0.029) |
| VRST to FLP | 0.006 ($5.1e^{-10}$-0.039) | 0.004 ($3.9e^{-09}$-0.028) | 0.003 ($7.7e^{-11}$-0.019) |
| VRST to FUT | 0.061 (0.003-0.145) | 0.006 ($5.6e^{-07}$-0.035) | 0.006 ($1.0e^{-06}$-0.035) |
| LLP to MLP | 0.005 ($4.3e^{-07}$-0.030) | 0.017 ($9.1e^{-06}$-0.075) | 0.002 ($9.0e^{-11}$-0.017) |
| LLP to MUT | 0.006 ($2.9e^{-06}$-0.032) | 0.006 ($9.1e^{-07}$-0.031) | 0.006 ($6.5e^{-06}$-0.030) |
| LLP to VRLP | 0.002 ($6.2e^{-11}$-0.013) | 0.002 ($2.5e^{-11}$-0.015) | 0.002 ($2.3e^{-11}$-0.018) |
| LLP to VRST | 0.002 ($1.1e^{-10}$-0.015) | 0.006 ($8.6e^{-07}$-0.032) | 0.006 ($9.7e^{-07}$-0.031) |
| LLP to LLT | 0.062 (0.016-0.124) | 0.288 (0.232-0.324) | 0.286 (0.223-0.325) |
| LLP to FLP | 0.005 ($2.1e^{-09}$-0.038) | 0.006 ($4.2e^{-09}$-0.038) | 0.010 ($2.3e^{-09}$-0.050) |
| LLP to FUT | 0.081 (0.026-0.149) | 0.006 ($9.6e^{-07}$-0.035) | 0.020 ($2.0e^{-05}$-0.078) |
| LLT to MLP | 0.006 ($5.7e^{-07}$-0.035) | 0.016 ($6.4e^{-06}$-0.076) | 0.002 ($2.7e^{-11}$-0.019) |
| LLT to MUT | 0.006 ($2.3e^{-06}$-0.037) | 0.005 ($1.0e^{-06}$-0.029) | 0.007 ($8.3e^{-06}$-0.031) |
| LLT to VRLP | 0.002 ($2.0e^{-11}$-0.015) | 0.002 ($3.8e^{-11}$-0.017) | 0.003 ($4.2e^{-11}$-0.019) |
| LLT to VRST | 0.003 ($9.9e^{-11}$-0.019) | 0.005 ($1.3e^{-06}$-0.028) | 0.006 ($1.1e^{-06}$-0.032) |
| LLT to LLP | 0.006 ($1.0e^{-06}$-0.036) | 0.001 ($1.0e^{-11}$-0.012) | 0.002 ($3.2e^{-12}$-0.016) |
| LLT to FLP | 0.003 ($1.8e^{-10}$-0.021) | 0.004 ($4.3e^{-08}$-0.031) | 0.003 ($1.4e^{-10}$-0.018) |
| LLT to FUT | 0.009 (0.001-0.034) | 0.006 ($6.9e^{-07}$-0.031) | 0.006 ($6.4e^{-07}$-0.029) |
| FLP to MLP | 0.007 ($8.4e^{-07}$-0.043) | 0.017 ($1.2e^{-05}$-0.078) | 0.002 ($4.3e^{-11}$-0.017) |
| FLP to MUT | 0.023 ($5.9e^{-06}$-0.087) | 0.005 ($2.0e^{-06}$-0.029) | 0.007 ($4.4\ e^{-06}$-0.032) |
| FLP to VRLP | 0.003 ($6.3e^{-11}$-0.022) | 0.002 ($1.8e^{-11}$-0.016) | 0.003 ($3.3e^{-10}$-0.017) |
| FLP to VRST | 0.003 ($2.1e^{-10}$-0.022) | 0.005 ($1.6e^{-06}$-0.027) | 0.006 ($6.5e^{-07}$-0.035) |
| FLP to LLP | 0.168 (0.079-0.250) | 0.001 ($9.8e^{-12}$-0.012) | 0.003 ($3.2e^{-11}$-0.020) |
| FLP to LLT | 0.176 (0.091-0.262) | 0.006 ($1.5e^{-06}$-0.031) | 0.007 ($1.3e^{-06}$-0.037) |
| FLP to FUT | 0.093 (0.013-0.190) | 0.271 (0.194-0.321) | 0.272 (0.197-0.321) |
| FUT to MLP | 0.006 ($1.2e^{-06}$-0.035) | 0.017 ($9.7e^{-06}$-0.076) | 0.002 ($2.3e^{-11}$-0.018) |
| FUT to MUT | 0.006 ($9.0e^{-07}$-0.033) | 0.006 ($9.4e^{-07}$-0.030) | 0.007 ($1.8\ e^{-06}$-0.034) |
| FUT to VRLP | 0.002 ($1.5e^{-10}$-0.012) | 0.002 ($3.1e^{-12}$-0.017) | 0.002 ($4.3e^{-11}$-0.016) |
| FUT to VRST | 0.003 ($2.1e^{-11}$-0.021) | 0.005 ($8.1e^{-07}$-0.029) | 0.006 ($1.7e^{-06}$-0.031) |
| FUT to LLP | 0.007 ($4.9e^{-07}$-0.037) | 0.002 ($3.4e^{-11}$-0.016) | 0.002 ($4.0e^{-11}$-0.018) |
| FUT to LLT | 0.010 ($5.4e^{-05}$-0.042) | 0.005 ($6.5e^{-07}$-0.028) | 0.006 ($5.2e^{-07}$-0.036) |
| FUT to FLP | 0.004 ($1.3e^{-09}$-0.028) | 0.008 ($2.9e^{-09}$-0.050) | 0.003 ($6.4e^{-10}$-0.022) |

Gene flow estimates represent the mean migration rate and 95% confidence intervals (in parentheses) for a pair of populations.
